# A Rare Case of Gallstone Ileus: Bouveret Syndrome Presenting with Concurrent Gallstone Coleus

**DOI:** 10.1155/2020/8844199

**Published:** 2020-11-07

**Authors:** Sena Park, Janaka Balasooriya, Thembi Ncube

**Affiliations:** Department of General Surgery, The Canberra Hospital, Yamba Drive, Garran Australian Capital Territory 2605, Australia

## Abstract

**Background:**

Bouveret syndrome and gallstone coleus are two rare subsets of gallstone ileus. Bouveret syndrome involves a gastric outlet obstruction, whereas gallstone coleus involves an obstruction of the large intestine. Both of the conditions are caused by gallstones, which migrated from the gallbladder via the fistulae. Due to its rarity, only few cases were reported for each condition. The current case describes an even rarer case of Bouveret syndrome and gallstone coleus presenting together. The case report will hopefully provide better understanding of the disease presentation and hence, lead to early diagnosis and management.

**Case:**

Ms. B is an 86-year-old woman of Italian background who presented to our emergency department with worsening symptoms of bowel obstruction. Her past clinical history included Kaposi sarcoma, hypertension, osteoarthritis, and vitamin D deficiency with surgical history including caesarean section and tonsillectomy. On her imaging, she had two large gallstones, one in the proximal duodenum and one in the distal colon. It also showed gastric dilatation and gas in the gall bladder. She was subsequently diagnosed with Bouveret syndrome with concurrent gallstone coleus. The laparotomy revealed two points of gallstone obstruction at the first part of the duodenum and at the distal sigmoid colon. Her postoperative recovery was uncomplicated. She was discharged to the care of her family and followed up in the general surgery clinic.

**Conclusion:**

The current case report describes a unique presentation of Bouveret syndrome where an additional gallstone was found simultaneously in the sigmoid colon causing the obstruction. By introducing this novel case of having two different subsets of gallstone ileus simultaneously, there will be a better understanding of both conditions and hopefully improve our scope of practice.

## 1. Introduction

Bouveret syndrome was first described by Leon Bouveret in 1896, where he reported two patients with gastric outlet obstruction caused by gallstones [1]. The culprit gallstones travel from the gallbladder to the stomach or duodenum via a cholecystoenteric or choledocoenteric fistula and subsequently cause mechanical obstruction of the gastric outlet [2]. It is a rare subset of gallstone ileus, which itself accounts for only 1-4 percent of all mechanical bowel obstructions. Despite its high morbidity and mortality, its rarity and nonspecific signs make early diagnosis challenging [3]. Hence, it is pivotal that medical practitioners are aware of different manifestations of the disease, minimizing the risk of delaying or even missing the diagnosis. Previously, there have been multiple case reports on Bouveret syndrome. However, its presentation with concurrent gallstone ileus in another location of gastrointestinal tract has been extremely rare. Gallstone coleus is another rare subset of gallstone ileus, which describes gallstone obstruction of the large bowel [4]. In this report, we aim to introduce an unusual case of Bouveret syndrome presenting with a concurrent gallstone coleus and discuss management options.

## 2. Case

Ms. B is an 86-year-old woman who presented with a four-day history of obstipation, anorexia, nausea, and vomiting associated with worsening colicky abdominal pain and postprandial reflux. Her past medical history included Kaposi sarcoma, hypertension, osteoarthritis, and vitamin D deficiency. Although she had acute cholecystitis in the past, cholecystectomy had not been performed. Her surgical history included caesarean section through a lower abdominal midline incision and tonsillectomy. She lived alone and was independent with her daily activities of living.

On examination, all the vital signs were within the normal range. Her abdomen was distended and tender on palpation as well as percussion, particularly in the lower abdomen. There was also guarding in the same region. Bowel sounds were present. On initial laboratory evaluation, she had mildly elevated inflammatory markers with white cell count of 15.6 [4.0–11.0 × 10^9^/L], C-reactive protein (CRP) of 67 [<6.0 mg/L]. Her liver function tests showed bilirubin of 25 [2-20 *μ*mol], alanine aminotransferase (ALT) of 18 [<33 U/L], alkaline phosphatase (ALP) of 135 [30-110 U/L], and gamma-glutamyl transpeptidase (GGT) of 56 [<56 U/L], indicating an obstructive pattern of the liver enzyme derangements. Other laboratory abnormalities were creatinine of 99 [45-90 *μ*mol/L] and urea of 8.6 [2.5-7.5 mmol/L], indicating mild acute kidney injury in the context of her reduced food intake and dehydration. Her other serum electrolytes were normal. The urinalysis was unremarkable.

**Figure 1:**
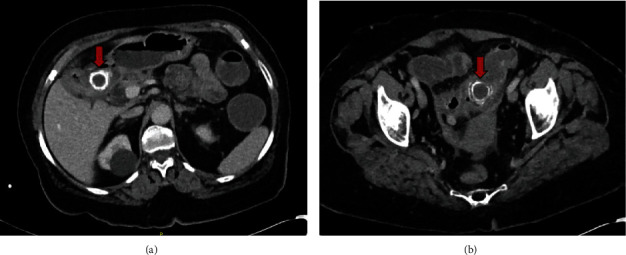
The first gallstone in the first part of the duodenum (a) and the second gallstone in the sigmoid colon (b) shown on axial CT scan.

**Figure 2:**
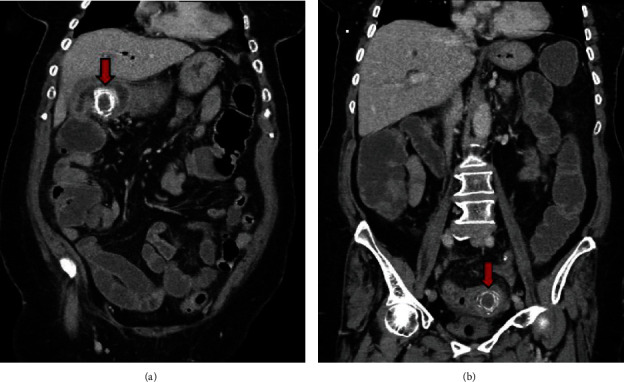
The first gallstone in the first part of the duodenum (a) and the second gallstone in the sigmoid colon (b) shown on coronal CT scan.

On her computed tomography (CT) scan of her abdomen and pelvis, there were two gall stones of 3 cm and 3.3 cm lodged in the proximal duodenum and distal colon, respectively (Figures 1 and 2). It also showed a contracted and inflamed gallbladder with gas in the biliary tree. Along with the gallstones, the presence of pneumobilia was consistent with the presence of fistula originating from the gallbladder. The common bile duct (CBD) was dilated, measuring 17 mm. The diagnosis of Bouveret syndrome with gallstone coleus was made.

While resuscitating with intravenous fluids, a nasogastric tube was inserted. Intravenous antibiotics (Ampicillin, Gentamicin, and Metronidazole) were commenced. The patient was consented for a laparotomy for removal of the gall stones and a potential bowel resection with stoma formation. Laparoscopic approach was not considered due to its technical difficulty with manoeuvring the gallstones and the likely presence of intra-abdominal adhesions.

**Figure 3:**
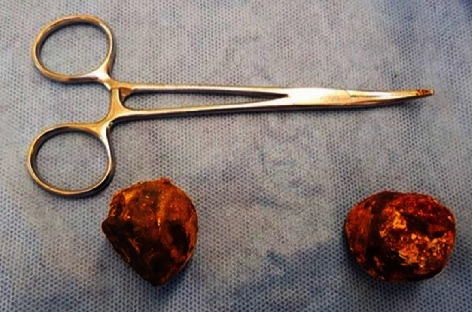
Two gall stones (left, 3 cm, and right, 3.3 cm) extracted from the proximal duodenum and sigmoid colon, respectively.

The laparotomy revealed two points of obstruction at the first part of the duodenum and at the distal sigmoid colon (Figure 3). An attempt was made to manually move the stone in the duodenum into the distended stomach but was unsuccessful. Following a duodenotomy, the duodenal stone was extracted. The sigmoid colon was distended proximal to the second stone. An indurated segment of the sigmoid colon was found distal to the stone. The cholecystocolonic fistula was not found. Similarly, a colotomy was made to remove the stone. There were no intraluminal pathology including strictures and masses identified. Both of the gallstones were removed via longitudinal incisions. The duodenotomy and colotomy were primarily closed transversely using PDS in a single layer. Duodenal repair was reinforced with an omental patch. There were severe inflammatory changes in the right upper quadrant involving the gall bladder and the second part of the duodenum, which were more in favour of a cholecystoduodenal fistula over a choledocoduodenal fistula.

On examination of the rest of the bowel, it was grossly normal except for multiple diverticulae in the left-sided large bowel. They were relatively small and uncomplicated diverticulae. The cause of the dilated CBD was not found. The cholecystectomy, resection of the fistula, and CBD exploration were not performed due to the unclear anatomy intraoperatively as well as patient comorbidities, making the risk of complications high.

Her immediate postoperative recovery was uncomplicated. She did not require inotropic or ventilator support. Her diet was slowly progressed. The patient was subsequently discharged to the care of her family and followed up in the general surgery clinic.

## 3. Discussion

There have been a number of cases reported on Bouveret syndrome, all describing its presentation with one gallstone causing the obstruction of the gastric outlet. However, the current case introduces a unique presentation of Bouveret syndrome with a simultaneous gallstone coleus, indicating two concurrent locations of obstruction in the gastrointestinal tract. Such presentation of Bouveret syndrome has not been reported previously, highlighting the novelty of the current case and its usefulness in understanding the disease.

Despite improvement in management strategies, the morbidity and mortality of Bouveret syndrome remain high at up to 60% and 30%, respectively. This has mainly been attributed to the fact that the majority of patients affected are elderly and have pre-existing comorbidities [5]. Therefore, early suspicion and diagnosis are crucial in Bouveret syndrome. Common presenting symptoms include nausea and vomiting and abdominal pain, which are found in approximately 85% and 70% of patients with Bouveret syndrome, respectively. Clinical signs of Bouveret syndrome include abdominal distension and tenderness and dehydration [6]. All of the symptoms and signs were present in our patient.

Imaging plays a critical role in diagnosing Bouveret syndrome. Rigler's triad includes gastric dilatation, a gallstone, and pneumobilia and represents imaging findings of Bouveret syndrome. Commonly used imaging modalities include CT and magnetic resonance cholangiopancreatography (MRCP), both of which have better sensitivity and specificity for Rigler's triad when compared with plain abdominal X-ray [7]. CT is often the preferred imaging modality in diagnosing Bouveret syndrome due to its wider availability than MRCP, and its superiority in detecting pneumobilia and impacted ectopic gallstones. MRCP is particularly useful to assess the cholecystoenteric fistula due its high sensitivity to delineate fluid in the fistula from the surrounding structures [8].

Bouveret syndrome is a subset of gallstone ileus, involving the gastric outlet or proximal duodenum. In fact, the most common point of obstruction in gallstone ileus is the terminal ileum, accounting for 50-90% of the cases. Less commonly, the obstruction is found in the jejunum in 20-40%, colon in 3-25%, and the duodenum and stomach in 1-10% [9]. A small series of cases in 1990 by Clavien et al. reported multiple gallstones in up to 16% of cases [10]. Hence, it is an essential practice that a surgeon looks for the presence of additional stones at the common sites of the obstruction, both on imaging and intraoperatively. Intraoperatively, the operating surgeon should ensure that there are no other stones by palpating the entire small and large bowels.

Endoscopic removal of gallstones in the intestine has been previously described and demonstrated reasonable outcomes in high-risk patients. Unfortunately, this approach is technically challenging, requires specialized resources, and often limited to smaller gallstones (less than 3 cm) [11]. Up to 91% of endoscopic and percutaneous attempts to extract the stones are unsuccessful, requiring surgery eventually [2]. Due to the clinical urgency reflected in our patient's presentation, difficult logistics in arranging the specialised endoscopic equipment and the unusual presence of two concurrent stones, we preferred the safest and quickest option of the open surgery. The surgical treatment as the first line intervention for the current presentation has resulted in the satisfactory recovery of the patient. Common surgical incisions for Bouveret syndrome include duodenotomy, pylorotomy, gastrotomy, or incision immediately proximal to the site of obstruction [12]. If feasible, the surgeon can manoeuvre the obstructing gallstone in the duodenum into the stomach to utilise gastrotomy for stone removal [13].

The colon is a rare location of obstruction in the gallstone ileus, accounting for 2% of all cases [14]. The relatively large diameter of the colon explains the rarity of the disease. The gallstone usually travels through a cholecysto-colonic fistula and subsequently impacts the bowel distally. A pathological narrowing commonly caused by diverticular pathology or pelvic irradiation can precipitate the impaction [15]. The sigmoid colon is the most common site of obstruction in the gallstone coleus and is often associated with a diverticular stricture [16]. Generally, a gallstone in the colon requires surgical removal as only 7% of patients achieve spontaneous passage of the stone [17]. Although there have been cases where extraction of stones was successful with colonoscopy, the colotomy remains as a mainstay treatment for large stones [18]. In the current case, we did not find any obvious strictures in the sigmoid colon. There was an indurated segment of sigmoid colon distal to the point of obstruction. This could account for the limitation of the distal bowel distension, preventing the stone moving forward towards the rectum.

In our case, both the duodenotomy and sigmoidotomy/colotomy were performed to extract the stones and transverse closures were used to prevent strictures. The surgical treatment has resulted in the satisfactory recovery of the patient. From our experience, we emphasise that the whole intestine is examined before and during the operation to ensure that there are no other gallstones present at another location. This will lower the risk of missing additional gallstone present in the gastrointestinal tract. Our experience in managing the unusual presentation of Bouveret syndrome hopefully contributes to the development of improved treatment strategies for the condition.

## 4. Conclusion

The current case report describes a unique presentation of Bouveret syndrome where an additional gallstone was found simultaneously in the sigmoid colon causing obstruction. Surgical treatment remains as the mainstay treatment modality in bowel obstructions due to gallstones. The current case emphasises the operative strategy of examining the entire bowel in a gallstone disease. Its rarity often makes early diagnosis and subsequent management challenging. However, it is important to understand different presentations of the disease and have a low threshold to investigate due to its high mortality and morbidity if left untreated.
